# Effect of Prior Chilling Period and Alga-Extract Packaging on the Quality of a Canned Underutilised Fish Species

**DOI:** 10.3390/foods9091333

**Published:** 2020-09-21

**Authors:** Santiago P. Aubourg, Marcos Trigo, Beatriz Martínez, Alicia Rodríguez

**Affiliations:** 1 Department of Food Technology, Marine Research Institute (CSIC), C/Eduardo Cabello, 6. 36208 Vigo, Spain; mtrigo@iim.csic.es; 2 Department of Food Technologies, CIFP Coroso, Avda. da Coruña, 174, 15960 Ribeira, Spain; bmartinezr@edu.xunta.gal; 3 Department of Food Science and Chemical Technology, Faculty of Chemical and Pharmaceutical Sciences, University of Chile, C/Santos Dumont 964, Santiago 8380000, Chile; arodrigm@uchile.cl

**Keywords:** *Scomber colias*, prior chilling, *Fucus spiralis*, packaging medium, canning, lipid damage, colour, trimethylamine, quality

## Abstract

The effect of a prior chilling period and an alga extract packaging on the quality of a canned underutilised mackerel species (*Scomber colias*) was investigated. For this different chilling times (0, 4 and 9 days) were taken into account and three concentrations of aqueous extracts of the macroalga *Fucus spiralis* were tested in a brine-packaging medium. Chemical changes related to quality were analysed after 3 months of canned storage. A substantial increase (*p* < 0.05) in free fatty acid content was observed in canned fish by increasing the chilling time; however, alga extract presence in the packaging medium led to decreased mean values. Concerning lipid oxidation development, an increased chilling time led to higher values (*p* < 0.05) of thiobarbituric acid index and fluorescent compounds formation; remarkably, an increased presence of alga extract led to a higher (*p* < 0.05) peroxide retention and lower (*p* < 0.05) fluorescent compounds content. Average colour L* and a* values showed a decrease and an increase, respectively, with chilling time; however, such changes were minimised with the alga extract content in the packaging system. Trimethylamine content revealed a marked increase as a result of the sterilisation step, but no influence (*p* > 0.05) of the chilling time or the alga-packaging medium could be implied.

## 1. Introduction

Canning represents one of the most important and traditional means of marine species preservation [1,2]. According to the thermal sensitivity of a broad number of constituents included in marine species, different kinds of detrimental effects have been reported, especially if over processing is carried out [3,4]. Among them, oxidation of lipids and vitamins and leaching of water-soluble vitamins and minerals can be mentioned. Contrary to other muscle food, marine species are generally caught or harvested in distant locations, so that the time elapsed till arrival to cannery can be a decisive period for the quality of the final product. Consequently, canneries are in the need of storing the raw material before it is canned or just transported to factory. With this purpose, two strategies have been employed abundantly, namely frozen and chilled storage. As a result, quality of canned marine products will strongly depend on the adequacy of storage times and temperatures employed to hold the raw material [5,6].

For centuries, marine algae have been included in the Asian diet, especially in countries like China, Japan and Korea. They have revealed to be an important source of beneficial constituents such as vitamins, trace minerals, dietary fibre, amino acids and unsaturated lipids [7]. Remarkably, algae are known to be exposed to a combination of high oxygen concentration and light. The lack of structural damage in their organs has led to the consideration that their protection against damage arises from their content on preservative substances [8]. Consequently, marine algae (brown, red and green) are attracting a great interest as a source of bioactive molecules such as polyphenols, alkaloids, terpenes, phycocyannins and carotenoids, all of them showing antioxidant activity [9,10]. According to the European Council regulation [11], algae are considered food or food ingredients, so that their use in food technology in general should not constitute any hazard to health. In spite of such advantages, algae use in foods as preservatives can be limited because of flavour, odour and colour considerations since effective preservative doses may exceed sensorial acceptable limits.

Among brown macroalgae, *Fucus spiralis* is an abundant species living in the Atlantic coasts of North America and Europe. This macroalga has attracted a great attention because of its valuable nutrition content [12,13] and the presence of different kinds of bioactive constituents [14,15,16], with reported antioxidant and antimicrobial activity during fish refrigeration [17], chilling [18] and canning [19].

Availability of traditional species is being constantly reduced, so that the search for unconventional sources has turned necessary for the fish industry [20,21]. Thus, small pelagic fish species represent a promising valuable and economic choice in different geographic areas. One such under-valued species is Atlantic chub mackerel (*Scomber colias*) [22,23], abundantly found in the Mediterranean Sea, the Atlantic Ocean and the Black Sea. Concerning its technological aptitude, its quality loss evolution was studied as a result of freezing [24,25], refrigerated storage under modified atmosphere and vacuum packaging [26], chilling storage [27] and canning [19].

In this study, the effect of a prior chilling period and the presence of a *F. spiralis* extract in the packaging medium employed for canning was investigated in canned chub mackerel quality. Quality analyses (lipid damage, colour changes and trimethylamine content) were carried out in mackerel muscle after 3 months of canned storage. These indices were chosen as being important markers of fish quality changes during the technological processes encountered (chilling storage and canning).

## 2. Materials and Methods

### 2.1. Initial Raw Fish and Chilling Storage

Specimens (130 fish) of fresh mackerel (length and weight ranges: 24.5–28.0 cm and 157–175 g, respectively) were obtained at Vigo harbour (North-Western Spain) in November 2017. Once at the laboratory, 10 fish individuals were taken and divided into five groups (two individuals per group). This fish (initial raw fish) were beheaded, eviscerated and filleted. Then, the white muscle was separated, pooled together within each group, minced and analysed independently (*n* = 5).

From the remaining whole fish individuals, 40 of them were immediately taken for the canning process (0 days of chilling time; day-0 samples). On the other side, 80 whole fish specimens were surrounded by ice at a 1:1 fish-to-ice ratio and placed in a small (2 m × 2 m, 2.5 m height) refrigerated room (4 °C). After 4 and 9 days of chilling storage, specimens (40 at each sampling time) were taken for the canning analysis. Storage temperature of fish specimens was +0.5 °C throughout the storage period. Boxes employed allowed draining, ice being renewed when required.

### 2.2. Preparation of Alga Extract

Lyophilised alga *F. spiralis* was obtained from Porto-Muiños (Cerceda, A Coruña, Spain). A mixture of 14 g of alga and 140 mL of distilled water was submitted to stirring for 30 s, sonication for 30 s and centrifugation at 3500× *g* for 30 min at 4 °C. The supernatant was then recovered and the extraction process was repeated. Supernatants were finally pooled together, made up to 250 mL with distilled water and then employed in the preparation of the packaging medium during the canning process, as expressed in the following sub-section.

### 2.3. Canning Process and Sampling Procedure

As previously indicated, the canning process was carried out on fish that was previously chilled for 0, 4 and 9 days. At each canning time, fish (40 whole fish individuals) were divided into 5 groups (8 specimens in each group). Then, fish were beheaded, eviscerated, filleted and 45 g pieces of mackerel fillets were introduced in flat rectangular cans (105 × 60 × 25 mm; 150 mL). Such fish portions included skin and whole muscle (i.e., white and dark muscles). Each can was prepared from one fish individual, so that eight cans were prepared in each group. For the packaging medium, 0, 5, 15 or 30 mL of the above-mentioned alga extract (corresponding to 0.00, 0.28, 0.84 and 1.68 g of lyophilised alga, respectively) were added to the cans and labelled as C-CT (canned control), C-F1 (low *F. spiralis* concentration), C-F2 (medium *F. spiralis* concentration) and C-F3 (high *F. spiralis* concentration) batches, respectively, followed by the addition of 40, 35, 25 or 10 mL of distilled water, respectively. Then, a brine solution (40 mL; 4% *w*/*v*) was added to each can, so that a final content of 0.00, 3.50, 10.50 and 21.00 mg extracted alga mL^−1^ packaging system was attained, respectively. At each canning time and within each group, two cans were prepared having the same alga extract content.

Contents of alga extract employed were based on preliminary trials. A concentration of 21.00 mg mL^−1^ packaging medium showed to be the highest concentration without modifying the flesh odour, colour and flavour of canned fish. Thus, this concentration was considered in the C-F3 batch, together with two less concentrated batches (C-F1 and CF-2).

After vacuum sealing, all cans were subjected to heat sterilisation treatment in a steam retort at 115 °C for 45 min (*F_o_* = 7 min) at the CIFP Coroso (Ribeira, A Coruña, Spain). After completing the heating time, the steam was cut off, the remaining steam was flushed away by air employment, and cans were cooled at reduced pressure. After a 3-month storage at 20 °C, the cans were opened and the liquid part was carefully drained off gravimetrically. Then, the fish white muscle was separated, wrapped in filter paper, and used for analysis. At each canning time, the fish white muscle of two cans with the same alga extract content was pooled together, minced and employed to carry out the different quality analyses. Each batch (C-CT, C-F1, C-F2 or C-F3) was analysed by means of five replicates (*n* = 5).

Solvents and chemical reagents used were in all cases reagent grade (Merck, Darmstadt, Germany).

### 2.4. Determination of Lipid Damage

Lipids were obtained by extraction of the mackerel white muscle by applying the Bligh and Dyer [28] method, which employs a chloroform-methanol (1:1) mixture. Lipid content is expressed as g lipid·kg^−1^ muscle.

Assessment of the free fatty acids (FFA) content was carried out on the muscle lipid extract according to Lowry and Tinsley [29]; this method is based on a complex formation with cupric acetate-pyridine followed by spectrophotometric determination at 715 nm (Beckman Coulter DU 640 spectrophotometer, Beckman Coulter Inc., Brea, CA, USA). Results are expressed as g FFA·kg^−1^ muscle.

Peroxide value (PV) was determined spectrophotometrically (520 nm) on the lipid extract by peroxide reduction with ferric thiocyanate [30]. Results are expressed as meq. active oxygen·kg^−1^ lipids.

Thiobarbituric acid index (TBA-i) was assessed according to the Vyncke [31] procedure. This method is based on the reaction between a trichloracetic acid extract of the fish white muscle and thiobarbituric acid. The content of thiobarbituric acid reactive substances (TBARS) is spectrophotometrically measured (532 nm) and calculated from a standard curve prepared from commercial 1,1,3,3-Tetraethoxypropane (TEP). Results are expressed as mg malondialdehyde·kg^−1^ muscle.

The fluorescent compounds formation (LS 45 fluorimeter; Perkin Elmer España; Tres Cantos, Madrid, Spain) was determined in the lipid extract of the fish white muscle as described by Aubourg and Medina [32]. The relative fluorescence (RF) was calculated as follows: RF = *F/F_st_*, where *F* is the fluorescence measured at each excitation/emission wavelength pair (namely 393/463 nm and 327/415 nm) and *F_st_* is the fluorescence intensity of a quinine sulphate solution (1 µg·mL^−1^ in 0.05 M H_2_SO_4_) at the corresponding wavelength pair. Results are expressed as the fluorescence ratio (FR), which was calculated as the ratio between the two RF values: FR = RF_393/463 nm_/RF_327/415 nm_.

### 2.5. Determination of Colour Changes and Trimethylamine Content

A tristimulus HunterLab Labscan 2.0/45 colorimeter (HunterLab, Reston, VA, USA) was applied in order to carry out the instrumental colour analysis (CIE 1976). Colour scores corresponding to each sample were averaged over four measurements, which were taken by rotating the measuring head 90° among triplicate measurements per position.

Determination of trimethylamine (TMA)-nitrogen (TMA-N) values was carried out by employing the picrate spectrophotometric (410 nm) method [33]. For it, a 5%-trichloracetic acid extract of the fish white muscle (10 g 25 mL^−1^) was prepared. Results are expressed as mg TMA-N·kg^−1^ muscle.

### 2.6. Statistical Analysis

Chemical and physical values were subjected to the ANOVA method to explore differences resulting from the effect of the prior chilling storage period and alga concentration in the packaging medium. As expressed above, five replicates (*n* = 5) were considered throughout the study. The least-squares difference (LSD) method was used to perform the comparison of means. Analyses were carried out using the PASW Statistics v.18 software for Windows (SPSS Inc., Chicago, IL, USA); differences were considered significant for a confidence interval at the 95% level (*p* < 0.05).

## 3. Results and Discussion

### 3.1. Determination of Lipid Hydrolysis Development

**Figure 1:**
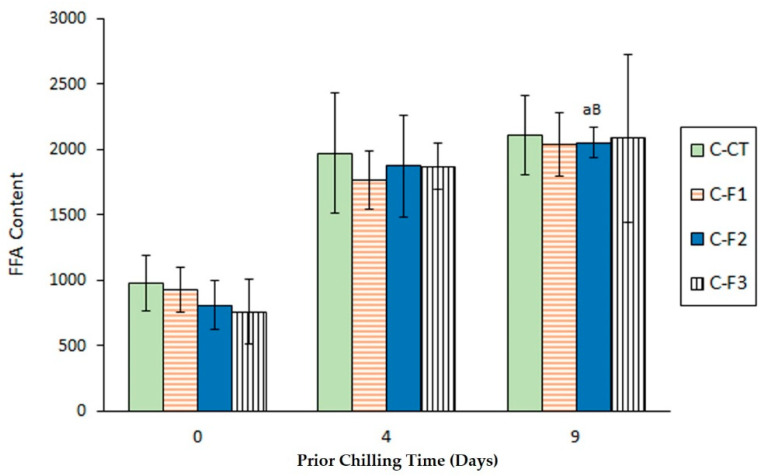
Assessment of free fatty acids (FFA) value (g·kg^−1^ muscle) in canned mackerel previously subjected to different chilling times and packaged with different alga extract concentrations. Mean values of five replicates (*n* = 5). Standard deviations are indicated by bars. Initial raw fish value: 37.15 ± 11.24. For each chilling time, the same low-case letter (a) denotes that no significant differences (*p* > 0.05) were obtained related to the alga extract presence. For each alga extract concentration, different capital letters (A, B) denote significant differences (*p* < 0.05) by effect of the chilling time. Alga extract concentrations in packaging media: C-CT (control), C-F1 (low alga content), C-F2 (medium alga content) and C-F3 (high alga content), in agreement with the Material and Methods section.

Compared with the initial raw fish, all kinds of canned samples corresponding to a 0-day chilling time showed a substantial increase (*p* < 0.05) of FFA content (Figure 1).

Furthermore, an additional general increase (*p* < 0.05) was also observed in canned fish by increasing the chilling time up to 4 days; contrary, a chilling storage extension up to 9 days did not lead to significant differences (*p* > 0.05) in the FFA content. Concerning the effect of the alga extract in the packaging medium, average FFA values showed a decreasing effect of *F. spiralis* extract content when considering samples corresponding to day-0 chilling time. If a 4 or 9-day chilling period is considered, average values corresponding to control canned fish (C-CT batch) were higher than their counterparts from the alga- packaging batches (C-F1, C-F2 and C-F3 batches); however, average values did not show a decreasing effect by increasing the alga extract presence.

FFA are considered to be the result of hydrolysis of high-molecular-weight lipid compounds such as triacylglycerols (TG) and phospholipids (PL). In the current research, FFA content can be considered the result of different factors. First, their formation during chilling storage should increase with chilling time by action of endogenous and microbial enzymes (phospholipases and lipases in general) [5,34]. Furthermore, the sterilization process can lead to hydrolysis of lipid classes such as TG and PL [6]. Interestingly, FFA are known to be rapidly oxidised by heating according to the fact that they provide a greater accessibility to oxygen and other oxidants in general when compared with TG and PL [35]. Finally, preservative compounds present in the alga extract can protect FFA from their breakdown during the heating process. On the basis of the data obtained, it can be concluded that an important effect of prior chilling time was implied, while the possible preserving effect of alga extract was not especially important.

In agreement with the current results, sunflower- or brine-packaged sprat (*Clupeonella cultriventris*) [36], as well as sunflower oil-packaged canned albacore tuna (*Thunnus alalunga*) subjected to different sterilisation conditions [37] revealed a marked FFA formation when compared with the starting raw material. Contrary to the present study, increasing the prior chilling period in brine-canned sardine (*Sardina pilchardus*) [32] and in sunflower oil-canned salmon (*Oncorhynchus kisutch*) [38] led to marked increases of the FFA content.

According to the present study, no effect of the alga extract concentration was produced on the FFA content of canned Atlantic mackerel (*Scomber scombrus*) packaged with an aqueous extract of macroalga *Bifurcaria bifurcata* [39]. Contrary, an increased formation of FFA was implied in canned Atlantic chub mackerel (*S. colias*) by increasing the presence of macroalgae *Ulva lactuca* and *F. spiralis* extracts in the packaging system [19].

### 3.2. Determination of Lipid Oxidation Development

Lipid oxidation development was measured by assessing different and complementary indices in order to obtain a satisfactory overview on the advance of this damage mechanism.

Canned samples corresponding to all chilling conditions showed a marked PV increase (*p* < 0.05) when compared with the initial raw fish (Table 1). Canned fish corresponding to control batch showed a progressive increase (*p* < 0.05) with chilling time, while alga-packaged fish did not provide differences (*p* > 0.05) as a result of the chilling time period. For all chilling times considered, peroxides content showed to be higher (*p* < 0.05) by increasing the alga extract content in the packaging medium. Thus, canned fish corresponding to C-F2 and C-F3 batches showed significant differences (*p* < 0.05) when compared with their counterpart control.

A significant formation of TBARS was not detected (*p* > 0.05) in canned fish subjected previously to a 0-day chilling in any of the batches under study (Table 1). However, if a 4-day storage was applied, a general increase was obtained in all batches, differences being significant (*p* < 0.05) in all alga-treated batches. Contrary, no significant differences (*p* > 0.05) were implied by increasing the chilling time up to 9 days; in this case, average values provided an increase in control batch, while a decrease was detected in canned batches including any alga extract concentration. A definite trend could not be concluded related to the alga extract presence in the packaging system. Thus, control canned fish showed higher average values after 0 and 9 days of chilling storage, while this batch showed the lowest average value when a 4-day storage is taken into account.

Comparison between initial raw fish and canned fish corresponding to day-0 storage showed an important fluorescent compounds formation (*p* < 0.05) as a result of the sterilisation step in all batches (Table 1). Furthermore, a progressive FR increase (*p* < 0.05) was produced in all batches by increasing the chilling time. This FR increase would be the result of interaction between primary and secondary lipid oxidation compounds (electrophilic behaviour) and food constituents possessing nucleophilic functions [32,40]. Remarkably, an inhibitory effect on fluorescent compounds formation was observed by the alga extract presence in the packaging medium. Thus, considering the canned fish corresponding to the 0-day time, all alga concentrations tested led to lower (*p* < 0.05) levels than the control; additionally, lower levels (*p* < 0.05) were observed in C-F3-batch canned fish when compared with their counterparts if a 9-day storage is taken into account.

Lipid oxidation is considered a complex deteriorative mechanism since it involves the formation of a wide range of molecules, most of them unstable, and consequently, able to breakdown and give rise to lower-weight compounds susceptible to react with nucleophilic-type molecules (proteins, peptides, free amino acids, etc.) present in fish muscle. As expressed above, this would be the case of peroxides and TBARS, widely reported to breakdown and give rise to tertiary (or interaction compounds) lipid oxidation compounds [32,40]. Since endogenous enzymes and microbial development are inactivated by heat, most attention in canned fish has been accorded to lipid oxidation and further interaction of oxidised lipids with other constituents, proteins especially, in agreement with their heat denaturation and consequently turning into more reactive molecules. Lipid damage development may be especially important if a fatty fish species is encountered as in the current study (lipid content: 74.3 ± 14.5 g kg^−1^ white muscle).

On the basis of the strong processing included in canning (i.e., sterilisation step), previous research has shown that assessment of primary and secondary lipid oxidation products does not lead to a definite trend about lipid quality changes in different kinds of canned fish such as oil- packaged canned albacore tuna (*T. alalunga*) sterilised under different conditions [37], as well as in other pelagic fish species such as olive-oil packaged bluefin tuna (*Thunnus thynnus*) and tomato sauce-packaged sardine (*Sardina pilchardus*) [41]. Indeed, the TBA-i led to a decreasing value in brine-canned sardine (*S. pilchardus*) by increasing the prior chilling time [32]. Remarkably, and in agreement with the current data, fluorescent compounds formation has shown to be a valuable quality index in canned fish. Thus, a progressive formation of fluorescent compounds was implied by increasing the chilling time in brine-canned sardine (*S. pilchardus*) [32] as well as in fish muscle and packaging system in sunflower-packaged canned sardine (*S. pilchardus*) [42].

In agreement with the current study, previous research has shown an increasing effect on peroxides content in canned fish by increasing the *B. bifurcata* water-extract [39] or the *U. lactuca* and *F. spiralis* brine-extract [19] contents in the packaging media employed for mackerel canning. In both studies, it was considered that peroxides breakdown was partially avoided as a result of the algae extract presence. Also in agreement with the current study, the formation of fluorescent compounds during canning was partially inhibited by increasing the macroalga extract presence both in water- [39] and brine-packaged [19] canned fish.

The antioxidant effect of alga *F. spiralis* extracts has already been proved in in vitro tests [12], showing a marked content on different kinds of antioxidant molecules such as polyphenols [15], alpha-tocopherol [13] and phlorotannins [14]. Antioxidant compounds, polyphenols especially, have been described as being able to stabilise free radical molecules so that lipid oxidation development would be inhibited and damage to other muscle constituents be decreased. Furthermore, water extracts obtained from macroalgae have shown to include different kinds of preserving phenolic acids (i.e., caffeic, chlorogenic, vanillic, etc.) [43], as well as sulphate polysaccharides, proteins, peptides, glycosides, low-molecular organic acids and salts) having potential preserving properties [44,45].

### 3.3. Determination of Colour Changes

**Figure 2:**
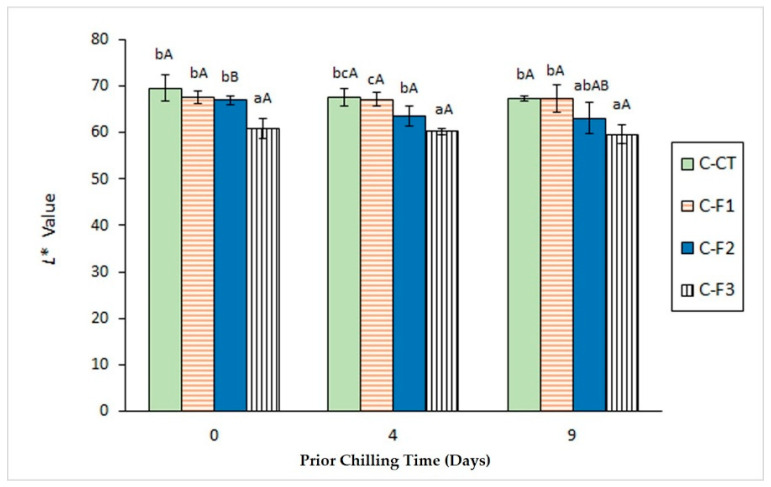
Determination of L* colour value in canned mackerel previously subjected to different chilling times and packaged with different alga extract concentrations. Mean values of five replicates (*n* = 5). Standard deviations are indicated by bars. Initial raw fish value: 38.30 ± 2.41. For each chilling time, different low-case letters (a, b, c) denote significant differences (*p* < 0.05) as a result of the alga extract concentration. For each alga extract concentration, different capital letters (A, B) denote significant differences (*p* < 0.05) by effect of the chilling time. Alga extract concentrations in packaging media as expressed in Figure 1.

**Figure 3:**
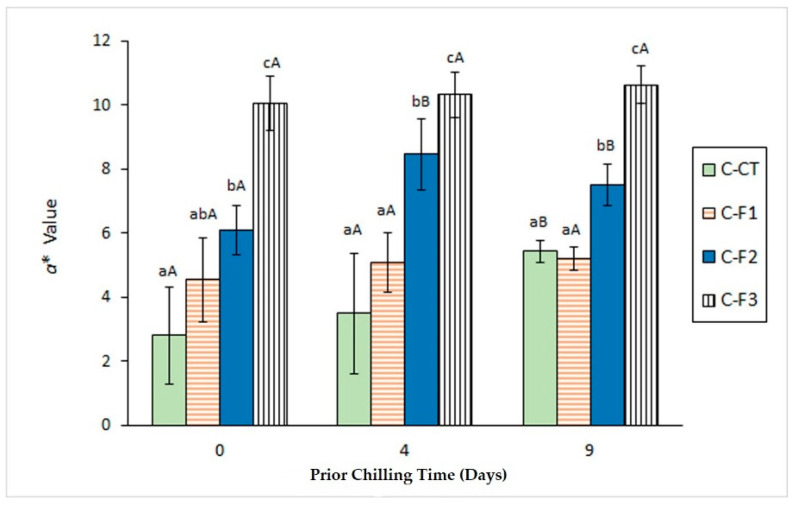
Determination of a* colour value in canned mackerel previously subjected to different chilling times and packaged with different alga extract concentrations. Mean values of five replicates (*n* = 5). Standard deviations are indicated by bars. Initial raw fish value: 15.56 ± 3.36. For each chilling time, different low-case letters (a, b, c) denote significant differences (*p* < 0.05) as a result of the alga extract concentration. For each alga extract concentration, different capital letters (A, B) denote significant differences (*p* < 0.05) related to the chilling time. Alga extract concentrations in packaging media as indicated in Figure 1.

Colour is considered as an important property in the appearance and acceptability of seafood by the consumer. Current data on colour assessment are depicted in Figure 2 and Figure 3. Taking into account the L* value of the initial raw fish, a general increase was observed as a result of canning, independently of the chilling period applied (Figure 2). Remarkably, no significant differences (*p* > 0.05) were practically obtained as a result of the chilling period, although the highest mean values were obtained in canned fish corresponding to the 0-day period in all batches. For all chilling times considered, decreasing L* values were obtained by increasing the alga extract concentration in the packaging system. Compared with control, significant differences (*p* < 0.05) were implied for canned fish including the most concentrated alga extract in canned samples corresponding to all storage times (0, 4 and 9 days).

Canning process led to a substantial decrease (*p* < 0.05) of a* value in all kinds of canned samples (Figure 3).

However, this decrease was partially inhibited (*p* < 0.05) by the presence of the alga extract in the packaging medium. Remarkably, an increase of the chilling time led to a progressive increase of average a* values in most batches, this increase being significant (*p* < 0.05) in fish corresponding to batches C-CT (9-day period) and C-F2 (4-day period). Concerning the effect of the alga-extract packaging, an increased value was detected for this colour parameter with the alga extract content in canned fish corresponding to all chilling times. It is concluded that the presence in the packaging system of preservative compounds (antioxidants, especially) from the alga extract has led to an inhibitory effect on the breakdown of molecules responsible for the a* value (carotenoids and pigments in general) in the mackerel muscle during the canning process.

Valuable results were not obtained by determination of the b* colour score; consequently, a definite effect of the chilling time and the alga extract presence in the packaging medium could not be observed for this parameter.

Previous related research has addressed the colour parameters assessment. Thus, Barbosa et al. [39] showed a marked increase for L* value in canned Atlantic mackerel (*S. scombrus*) by comparison with the initial raw fish. Remarkably, this increase was partially inhibited by increasing the concentration of an aqueous extract of macroalga *B. bifurcata* in the packaging system. Furthermore, increasing the previous storage temperature and time led to an increase in L* value and a decrease in a* value in canned skipjack tuna (*Katsuwonus pelamis*) [46] and coho salmon (*O. kisutch*) [38]. Indeed, an a* value decrease has been pointed out as being correlated with haemoglobin-mediated lipid oxidation in fish and to show an inverse relationship with the content on secondary lipid oxidation compounds [47,48].

### 3.4. Trimethylamine Content Assessment

Contents on TMA values are depicted in Table 2. Prior chilling storage did not lead to a definite effect on TMA content in canned fish. Thus, highest average values (*p* < 0.05) were obtained after 4 days in samples corresponding to C-CT and C-F3 batches, while a 9-day chilling period showed that canned fish from C-F1 and C-F2 batches revealed the highest levels (*p* < 0.05). Furthermore, a strong formation (*p* < 0.05) of TMA was observed after the sterilisation process in all kinds of canned samples. Concerning the effect of the alga extract in the packaging medium, some decreasing effect could be observed in TMA content in canned samples corresponding to a 0-day storage; however, canned samples related to 4 and 9 days of storage did not provide a definite trend (*p* > 0.05). Thus, higher mean TMA-N values were detected in canned control fish than in any other counterpart fish when a 0- and 4-day chilling period is considered. However, if the longest chilling time (i.e., 9 days) is taken into account, a higher mean value was obtained in C-F1-batch canned fish.

TMA formation in the present research can be justified as a result of two different pathways. One side, trimethylamine oxide (TMAO) can be broken down by bacterial hydrolysis during chilled storage [34]. On the other side, TMA can be produced by breakdown of TMAO and other muscle constituents during the sterilisation step [6]. Since a great difference between raw and day-0 canned samples was observed, the second effect has shown to be largely more important than the first one. Interestingly, as being a tertiary amine, TMA would not be leached into the brine-packaging system as not being a water-soluble compound. Since no effect of the alga extract presence in the packaging medium was perceived, it is concluded that alga preservative compounds did not alter the TMA formation during the sterilisation process.

Although including different packaging media than the current one, similar results on TMA values have been obtained previously in canned fish. Thus, Gallardo et al. [49] observed a marked formation of TMA as a result of cooking and sterilisation in oil-packaged canned albacore tuna (*T. alalunga*), while a marked loss of TMAO was detected in sunflower-packaged canned sardine (*S. pilchardus*) by increasing the prior chilling time [42]. A substantial formation of TMA was also detected in olive-oil packaged bluefin tuna (*T. thynnus*), tomato sauce-packaged sardine (*S. pilchardus*) muscle [41] and in sunflower oil-packaged canned salmon (*O. kisutch*) that was previously stored under traditional and slurry icing conditions [38]. Concerning the effect of the alga extract content in the packaging system, previous research has shown, as in the current research, no definite effect on the TMA content in canned mackerel species when a water- [39] or a brine-packaged [19] medium was employed.

## 4. Conclusions

This study focused on the quality of a canned fish product prepared from a fatty under-valued species. In it, the effect of a prior chilling period and the employment of a packaging system including an alga extract on different physico-chemical properties of canned chub mackerel were investigated. As a result, an increased chilling time led to a quality loss (*p* < 0.05) that was observed in lipid hydrolysis (FFA formation) and oxidation (TBA-i and FR determinations) development; remarkably, no effect (*p* > 0.05) was detected in TMA levels and colour parameters (L* and a*). On the other side, packaging including *F. spiralis* extracts medium led to an average decrease of lipid hydrolysis development (FFA content) and to a significant (*p* < 0.05) quality loss inhibition according to values obtained for the FR and colour parameters (L* and a*); furthermore, no effect (*p* > 0.05) of alga-packaging was detected in TMA content, while a higher retention (*p* < 0.05) of primary oxidation molecules (i.e., peroxides) was concluded by increasing the alga concentration.

Current results show the strong dependence of canned fish quality on the holding conditions of the raw material employed. Remarkably, a packaging system including a macroalga extract has been tested and found profitable to enhance the quality of a canned under-valued fish species. It is considered that the development of optimised conditions of this alga-packaging system may open the way to its application on all kinds of fish species, these including high-value fish such as tuna, bonito or salmon.

## Figures and Tables

**Table 1:** Determination of peroxide value (PV), thiobarbitruric acid index (TBA-i) and fluorescence ratio (FR) in canned mackerel packaged with different alga extract concentrations and previously subjected to different chilling times.

Quality Index	Alga Extract Concentration	Prior Chilling Time (Days)		
		**0**	**4**	**9**
PV (meq. active oxygen·kg^−1^ lipids)	C-CT	a 1.01 ± 0.24 A	a 1.31 ± 0.81 AB	a 2.14 ± 0.55 B
	C-F1	b 3.08 ± 0.91 A	ab 2.08 ± 0.72 A	ab 2.89 ± 0.57 A
	C-F2	b 3.68 ± 0.61 A	bc 3.01 ± 0.55 A	b 3.63 ± 0.53 A
	C-F3	c 6.88 ± 1.60 A	d 6.84 ± 1.47 A	c 6.50 ± 1.24 A
TBA-i (mg malondialdehyde·kg^−1^ muscle)	C-CT	a 0.73 ± 0.22 A	a 1.22 ± 0.40 AB	a 1.39 ± 0.40 B
	C-F1	a 0.51 ± 0.20 A	a 1.77 ± 0.65 B	a 1.28 ± 0.33 B
	C-F2	a 0.44 ± 0.07 A	a 1.69 ± 0.35 B	a 1.27 ± 0.45 B
	C-F3	a 0.48 ± 0.09 A	a 1.36 ± 0.39 B	a 1.02 ± 0.14 B
FR	C-CT	c 4.34 ± 0.57 A	a 5.27 ± 0.55 A	b 6.08 ± 0.14 B
	C-F1	ab 3.30 ± 0.16 A	a 5.06 ± 0.50 B	b 6.28 ± 0.31 C
	C-F2	b 3.56 ± 0.14 A	a 4.73 ± 0.10 B	b 6.03 ± 0.25 B
	C-F3	a 2.96 ± 0.33 A	a 4.99 ± 0.12 B	a 5.01 ± 0.36 B

Average values ± standard deviations of five replicates (*n* = 5). Initial raw fish values: 0.43 ± 0.31 (PV), 0.52 ± 0.24 (TBA-i) and 2.00 ± 0.47 (FR). For each chilling time, values preceded by different low-case letters (a, b, c, d) denote significant differences (*p* < 0.05) by effect of the alga extract concentration. For each alga extract concentration, values followed by different capital letters (A, B, C) denote significant differences (*p* < 0.05) related to the chilling time. Alga extract concentrations in packaging media: C-CT (control), C-F1 (low alga content), C-F2 (medium alga content) and C-F3 (high alga content), according to the Material and Methods section.

**Table 2:** Determination of trimethylamine-nitrogen (TMA-N) content in canned mackerel packaged with different alga extract concentrations and previously subjected to different chilling times.

Quality Index	Alga Extract Concentration	Prior Chilling Time (Days)		
		0	4	9
TMA-N (g·kg^−1^ muscle)	C-CT	a 25.41 ± 2.64 A	b 31.40 ± 3.79 A	ab 28.12 ± 6.10 A
	C-F1	a 23.78 ± 5.65 A	a 18.06 ± 3.26 A	b 33.58 ± 2.77 B
	C-F2	a 21.21 ± 4.22 A	a 18.55 ± 3.32 A	a 24.96 ± 5.11 A
	C-F3	a 21.70 ± 1.87 A	b 28.71 ± 2.05 B	a 26.40 ± 2.64 B

Mean values ± standard deviations of five replicates (*n* = 5). Initial raw fish value: 1.04 ± 0.12 (TMA-N). For each chilling time, values preceded by different low-case letters (a, b) denote significant differences (*p* < 0.05) related to the alga extract concentration. For each alga extract concentration, values followed by different capital letters (A, B) denote significant differences (*p* < 0.05) by effect of the chilling time. Alga extract concentrations in packaging media as indicated in Table 1.
